# Transmission of West Nile Virus by *Culex quinquefasciatus* Say Infected with Culex Flavivirus Izabal

**DOI:** 10.1371/journal.pntd.0000671

**Published:** 2010-05-04

**Authors:** Rebekah J. Kent, Mary B. Crabtree, Barry R. Miller

**Affiliations:** Division of Vector-Borne Infectious Diseases, Arbovirus Diseases Branch, Centers for Disease Control and Prevention, Fort Collins, Colorado, United States of America; University of Texas Medical Branch, United States of America

## Abstract

**Background:**

The natural history and potential impact of mosquito-specific flaviviruses on the transmission efficiency of West Nile virus (WNV) is unknown. The objective of this study was to determine whether or not prior infection with Culex flavivirus (CxFV) Izabal altered the vector competence of *Cx. quinquefasciatus* Say for transmission of a co-circulating strain of West Nile virus (WNV) from Guatemala.

**Methods and Findings:**

CxFV-negative *Culex quinquefasciatus* and those infected with CxFV Izabal by intrathoracic inoculation were administered WNV-infectious blood meals. Infection, dissemination, and transmission of WNV were measured by plaque titration on Vero cells of individual mosquito bodies, legs, or saliva, respectively, two weeks following WNV exposure. Additional groups of *Cx. quinquefasciatus* were intrathoracically inoculated with WNV alone or WNV+CxFV Izabal simultaneously, and saliva collected nine days post inoculation. Growth of WNV in *Aedes albopictus* C6/36 cells or *Cx. quinquefasciatus* was not inhibited by prior infection with CxFV Izabal. There was no significant difference in the vector competence of *Cx. quinquefasciatus* for WNV between mosquitoes uninfected or infected with CxFV Izabal across multiple WNV blood meal titers and two colonies of *Cx. quinquefasciatus* (p>0.05). However, significantly more *Cx. quinquefasciatus* from Honduras that were co-inoculated simultaneously with both viruses transmitted WNV than those inoculated with WNV alone (p = 0.0014). Co-inoculated mosquitoes that transmitted WNV also contained CxFV in their saliva, whereas mosquitoes inoculated with CxFV alone did not contain virus in their saliva.

**Conclusions:**

In the sequential infection experiments, prior infection with CxFV Izabal had no significant impact on WNV replication, infection, dissemination, or transmission by *Cx. quinquefasciatus*, however WNV transmission was enhanced in the Honduras colony when mosquitoes were inoculated simultaneously with both viruses.

## Author Summary

Unlike most known flaviviruses (Family, *Flaviviridae*: Genus, *Flavivirus*), insect-only flaviviruses are a unique group of flaviviruses that only infect invertebrates. The study of insect-only flaviviruses has increased in recent years due to the discovery and characterization of numerous novel flaviviruses from a diversity of mosquito species around the world. The widespread discovery of these viruses has prompted questions regarding flavivirus evolution and the potential impact of these viruses on the transmission of flaviviruses of public health importance such as WNV. Therefore, we tested the effect of Culex flavivirus Izabal (CxFV Izabal), an insect-only flavivirus isolated from *Culex quinquefasciatus* mosquitoes in Guatemala, on the growth and transmission of a strain of WNV isolated concurrently from the same mosquito species and location. Prior infection of C6/36 (*Aedes albopictus* mosquito) cells or *Cx. quinquefasciatus* with CxFV Izabal did not alter the replication kinetics of WNV, nor did it significantly affect WNV infection, dissemination, or transmission rates in two different colonies of mosquitoes that were fed blood meals containing varying concentrations of WNV. These data demonstrate that CxFV probably does not have a significant effect on WNV transmission efficiency in nature.

## Introduction

The majority of the >70 recognized flaviviruses (family *Flaviviridae*, genus *Flavivirus*) are arthropod-borne, and include some of the world's most historically- and medically-important viruses including Yellow fever (YFV) and the Dengue (DENV) viruses. Gaunt et al. [1] described four distinct evolutionary clades within the genus *Flavivirus* that correlated with geography, vector, and associated disease: tick-borne, *Culex*-borne, *Aedes*-borne, and no known vector. Basal to all of these groups was Cell fusing agent virus (CFAV), an insect-only flavivirus discovered in an *Aedes aegypti* cell line more than 30 years ago [2].

Recently, a number of novel flaviviruses which cluster phylogenetically with CFAV have been isolated and identified from a diversity of field-collected mosquitoes and ticks around the world, including known arbovirus vectors. These arthropod-specific viruses collectively represent a unique clade of flaviviruses and include Ngoye virus from *Rhipicephalus* ticks in Senegal [3], Kamiti River virus (KRV), isolated from *Aedes mcintoshi* Huang in Kenya [4], [5], CFAV isolated from *Ae. aegypti* in Thailand and Puerto Rico [6], [7], Quang Binh virus from *Culex tritaeniorhynchus* Giles in Vietnam [8], and Nounané virus from *Uranotaenia mashonaensis* Theobald in Côte d'Ivoire [9]. Additionally, many strains of Culex flavivirus (CxFV) have been isolated from *Culex pipiens* L. in Japan [10], and North America [11], *Culex tarsalis* Coquillett throughout the western United States and Canada [11]–[12] (Bolling et al., unpublished data), *Cx. restuans* Theobald from Texas [13], and *Cx. quinquefasciatus* Say from Guatemala [14], the Yucatan Peninsula [15], Texas and Trinidad [13]. While there has been extensive genetic characterization of these viruses, the natural history and potential impact of mosquito-specific flaviviruses on the transmission efficiency of arboviruses of public health importance such as West Nile virus (WNV) remains unclear.

Arbovirus superinfection in mosquitoes and mosquito cell culture has been previously studied [16]–[25]. Infection with one flavivirus has been shown to suppress infection and prevent transmission of a second, antigenically-similar flavivirus. This phenomenon was demonstrated for Japanese encephalitis virus and Murray Valley encephalitis (MVE) virus superinfections in *Culex tritaeniorhynchus* Giles [16], two different strains of WNV in *Culex pipiens* form molestus Forskal [17], and WNV and St. Louis encephalitis virus in *Cx. quinquefasciatus* [18]. Sabin [19] demonstrated that high doses of YFV administered to *Ae. aegypti* previously infected with DENV still resulted in transmission of YFV, although mosquitoes were less susceptible to secondary infection with YFV. Similar findings have been reported in cell culture, where homologous superinfections were inhibited but secondary infection with a heterologous virus was permitted [22]–[25]. Therefore, based on previous observations, a primary infection of mosquitoes with a mosquito-specific flavivirus has the potential to interfere with infection or transmission of WNV acquired secondarily.

West Nile virus activity has been documented in Guatemala since 2003, beginning with the detection of WNV seroconversions in horses [26]. Serological evidence of WNV transmission has since been found in wild birds and chickens (Morales-Betoulle et al., unpublished data) and WNV has been isolated from several species of *Culex (Culex)* mosquitoes including *Cx. quinquefasciatus* (Morales-Betoulle et al., unpublished data). *Culex quinquefasciatus* is abundant in the urban WNV transmission focus comprising the city of Puerto Barrios, Guatemala, however, there has been little evidence of WNV-associated human disease in Guatemala or elsewhere in Latin America [27]. CxFV Izabal strain has also been found co-circulating with WNV in *Cx. quinquefasciatus* in Guatemala [14]. Minimum infection rates of CxFV in *Cx. quinquefasciatus* in Latin America were 20.8 per 1000 in Mexico [15] and 4.7 per 1000 in Guatemala [14]. Prevalence of CxFV Izabal in *Cx. quinquefasciatus* and the potential for this mosquito-specific flavivirus to disrupt WNV transmission is one of several hypotheses that have been proposed to explain the lack of human disease attributable to WNV in Latin America [15].

The objective of this study was to determine if prior infection with CxFV Izabal altered the vector competence of *Cx. quinquefasciatus* for transmission of WNV. The specific aims of this work were to: 1) compare replication kinetics of a Guatemalan isolate of WNV in CxFV Izabal – infected (CxFV Izabal (+)) and CxFV Izabal-uninfected (CxFV Izabal (−)) C6/36 cells and female *Culex quinquefasciatus*, 2) compare infection, dissemination and transmission rates of WNV in *Cx. quinquefasciatus* either infected or uninfected with CxFV Izabal, 3) determine whether WNV transmission by CxFV Izabal (+) *Cx. quinquefasciatus* is influenced by WNV blood meal titer, mosquito colony, simultaneous inoculation with CxFV, or inoculation with heat-inactivated CxFV. These data test the null hypothesis that there is no difference between the replication or transmission of WNV in CxFV Izabal (+) and CxFV Izabal (−) cells or mosquitoes

## Materials and Methods

### Viruses, cells, and mosquitoes

All CxFV experiments utilized CxFV Izabal isolate GU-06-2692, passage 1, isolated from a pool of *Cx. quinquefasciatus* in Puerto Barrios, Guatemala, 2006 [14]. West Nile virus isolate GU-06-2256, passage 3, also isolated from *Cx. quinquefasciatus* in Puerto Barrios, Guatemala, was used for flavivirus co-infection and vector competence studies. The growth of CxFV Izabal in cell culture was compared to that of KRV, strain SR-75 [4], [5]. *Aedes albopictus* C6/36-ATCC cells (American Type Culture Collection, Manassas, VA) maintained at 28°C in were used for growth and plaque titration of CxFV Izabal, and Vero (African green monkey kidney) cells maintained at 37°C were used for WNV plaque titrations. Two strains of *Cx. quinquefasciatus* were used in this study. The Sebring colony was originally established from Florida in 1988 and has been in colony at the CDC in Fort Collins since 2004. In an attempt to utilize viruses and vectors from the same geographic region, *Cx. quinquefasciatus* from Tegucigalpa, Honduras were colonized in September 2008. Generations F5/6 and F12 of the Honduras colony were used in this study. Prior to use, both colonies were confirmed CxFV-negative by RT-PCR.

### Virus quantification

CxFV Izabal was quantified from cell culture supernatant and homogenized mosquitoes by plaque titration on C6/36 cells [28]. Plaque assays were performed on C6/36 cell monolayers in 6-well plates using a double overlay method in nutrient media (5× Earle's BSS, 6.6% yeast extract-lactalbumin hydrolysate, 6% sodium bicarbonate, 4% FBS, and 0.4% gentamycin) mixed 1∶1 with 2.6% low-melt Sea Plaque agarose. Second overlay containing neutral red was added at seven DPI. WNV was quantified by Vero cell plaque assay using the double overlay method [28]. Second overlay containing neutral red was added 2 DPI.

CxFV Izabal viral RNA was also quantified by qRT-PCR. RNA extractions were performed using the QIAamp Viral RNA Mini Kit according to manufacturer's instructions (Qiagen Inc., Valencia, CA) with an elution volume of 100 µl. Quantification of viral RNA from 10-fold dilutions of RNA extracted from 100 µl stock virus of known concentration was used for the qRT-PCR standard curve. Four qRT-PCR primer and probe sets were designed from NS5 and E gene regions of CxFV RNA using Primer Express software (Applied Biosystems Inc, Foster City, CA) (Table 1). The complete genome sequence of CxFV (GenBank Accession number NC_008604) [10] and available RNA sequence from CxFV Izabal (EU805805, EU805806) [14] were used to select primers. CxFV Izabal primer and probe sensitivity and specificity were evaluated by sequence comparison to CFAV and CxFV (Table 1), and by testing each primer and probe set on a dilution series of available isolates of CxFV Izabal, WNV and KRV [4], [5].

**Table 1:** qRT-PCR primers and probes used for detection and quantification of CxFV Izabal RNA.

Target	Primer name	5′ to 3′ sequence	Tm	Nucleotides different from CxFV from Japan (NC_008604)	Nucleotides different from CFAV (NC_001564)
E	E92F	CGCCGAACGGACTTCTTG	59	0	4
	E151R	TCCATTGGCCGCCATATATC	59	0	4
	E112Probe	TTCCGCACTGGAGCAGCCG	70	2	8
E	E232F	GGTGAGATCAACGGCAAAGAA	59	2	7
	E295R	CAGTTCCCCATCCACGATTG	59	0	4
	E295Probe	CGTTTGCTCAACCCAGCCCT	68	1	4
NS5	NS5372F	CCACACCAGTCTTCGGTACATC	58	2	8
	NS5438R	CGGTTCGGTAGGTTGCAAGT	59	3	11
	NS5395Probe	CTGCTGCGTCAAAAACGCGCAA	69	2	8
NS5	NS5618F	GGCTCACGCCCAGATGTG	60	3	3
	NS5688R	TGATGGCGGCGAATCC	59	2	5
	NS5638Probe	CGTTGTACTACTTCCATCGGAGAGATCTGCG	69	5	8

qRT-PCR assays were correlated to plaque titration on C6/36 cells. Ten-fold serial dilutions of CxFV Izabal were split such that RNA was extracted from 100 µl of each virus dilution for quantification by qRT-PCR, and the remaining sample was subjected to plaque titration on C6/36 cells as described above. RNA copies/mL determined for each virus concentration were plotted against the corresponding pfu/mL determined by C6/36 cell plaque titration.

### Antiserum preparation

All animals were handled in strict accordance with the standards and policies of the Department of Health and Human Services' Office of Laboratory Animal Welfare (OLAW) and the US Department of Agriculture's Animal Welfare Act. All animal work was approved by the Centers for Disease control Institutional Animal Care and Use Committee, Protocol # 06-011. Hyperimmune polyclonal antisera against CxFV Izabal was generated in Swiss-Webster mice. Twenty-five adult female mice were housed in groups of five animals per cage. Each of four groups of five mice was immunized intraperitoneally with 0.1 mL CxFV Izabal virus seed (infected C6/36 cell, passage 1, tissue culture supernatant) diluted either 1∶10 or 1∶100 in Dulbecco's phosphate buffered saline (PBS). Mice were administered boosters of the same virus stock and concentration 3 wks and 6 wks following the initial vaccination. The fifth group was sham-inoculated with 0.1 mL PBS. Hyperimmunized mice were bled out by cardiac puncture three weeks following the third immunization. Blood was collected directly into microtainer tubes and centrifuged for serum separation. Pooled and individual aliquots of hyperimmune sera were stored at −80°C.

Because some antibodies in the sera were found to bind to C6/36 cells during immunofluorescence assay (IFA), sera were 4× cross-adsorbed against sonicated C6/36 cells to remove C6/36-specific antibodies. Uninfected C6/36 cells in DMEM maintenance medium containing 2% FBS were harvested from a T25 flask and washed once with PBS. Washed cells were pelleted by centrifugation at 5000 rpm for 5 min at 4°C, and resuspended in 1 mL PBS. Aliquots of 50 µl cell suspension were transferred to 0.2 mL PCR tubes and sonicated for 5 min. Five hundred microliters of pooled sera was cross-adsorbed with 200 µl sonicated C6/36 cell suspension at room temperature for 1.5 hrs with continuous mixing. Cell debris was removed from adsorbed sera by centrifugation at 5000 rpm for 5 min. The supernatant was adsorbed three additional times as above using fresh sonicated cells. Clarified antiserum was stored at −20°C.

### Immunofluorescence assay

Immunofluorescence assay using polyclonal mouse anti-CxFV serum was optimized using slides spotted with CxFV Izabal (+) and CxFV Izabal (−) C6/36 cells. To generate infected cells for spot slides, a T25 flask was inoculated with CxFV Izabal at a multiplicity of infection (MOI) of 0.1. Virus was allowed to adsorb for 1 hour at 28°C in 1 mL DMEM with 2% FBS, rocking every 15 min. After one hour, the volume of medium was increased to 5 mL. Cells were harvested at 4 DPI, washed twice with cold PBS, and acetone-fixed to 12-well multispot slides for 10 min (Thermo Electron Corp., Pittsburgh, PA). Uninfected C6/36 cells were harvested and fixed to slides as negative control. Spot slides were incubated with serial 2-fold dilutions of polyclonal mouse anti-CxFV for 30 minutes at 37°C in a humid box. Slides were washed twice for 10 min in PBS and air dried. Slides were then incubated for 30 min at 37°C in a humid box with secondary antibody conjugate AlexaFluor 488 goat anti-mouse IgG H+L (Invitrogen, Molecular Probes, Eugene, OR), diluted 1∶1000 in PBS with 0.08% trypan blue. Again, slides were washed twice with PBS, rinsed briefly with distilled water, and air dried. Cover slips were mounted using SlowFade Gold mounting medium (Invitrogen, Molecular Probes, Eugene, OR) and visualized with a Zeiss AxioImager Z1 (Carl Zeiss Microimaging, Inc., Thornwood, NY).

For IFA on mosquito tissues, mosquitoes were dipped briefly in 70% EtOH to destroy hydrophobicity. Midguts and ovaries were dissected in PBS using fine forceps on a microscope slide. Dissected tissues were placed on poly-L-lysine-coated slides (Polysciences, Inc.Warrington, PA) and allowed to dry. Wells were drawn around each tissue using a TexPen plastic pen (Mark-Tex Corp., Englewood, NJ), and tissues were fixed in ice cold acetone for 10 min. Headsquashes were performed by squashing dissected heads directly onto clean spot slides with a coverslip and manually removing pieces of cuticle, followed by acetone fixation. Immunostaining of mosquito tissues was performed as described above. For staining of CxFV+WNV co-infected tissues, human anti-WNV IgG obtained from the CDC reference collection (CDC, DVBID, Fort Collins, CO) was used as a primary antibody in addition to the anti-CxFV serum. Both CxFV and WNV antisera were used at 1∶320 dilution. Secondary staining of co-infected tissues utilized AlexaFluor 594 goat anti-mouse IgG H+L and AlexaFluor 488 goat anti-human IgG H+L each diluted 1∶1000 in PBS/trypan blue.

### Virus growth curves

CxFV Izabal growth was measured in C6/36 cells and in *Cx. quinquefasciatus*. To measure virus growth in cell culture, C6/36 cells were inoculated with CxFV Izabal at an MOI of 0.03 or 0.1. Virus was allowed to adsorb for one hour at 28°C in 1 mL DMEM containing 2% FBS. After one hour, cells were washed three times with PBS, and 5 mL cell culture maintenance medium was added. One flask of no-virus control was maintained simultaneously. Supernatant aliquots were harvested from each flask at 0, 1, 2, 4, 6, 8, 10, 12, and 14 DPI and stored at −80°C. Samples were clarified by centrifugation and titrated by C6/36 cell plaque assay as described above.

For growth *in vivo*, groups of *Cx. quinquefasciatus* Sebring strain mosquitoes were infected with CxFV Izabal either by intrathoracic inoculation [29] or *per os*. For inoculations, approximately one week-old female *Cx. quinquefasciatus* were inoculated with 1.9 log_10_ pfu±1.6 log_10_ pfu CxFV Izabal. Mosquitoes were housed in screened paperboard pint containers held at 28°C and 95% relative humidity. Three to five mosquitoes were harvested on Days 0, 2, 4, 8, 12, and 14 post inoculation. For virus infection *per os*, C6/36 cells were inoculated with CxFV Izabal at an MOI of 0.1, as above. Virus-infected cell culture supernatant was harvested 4 DPI and clarified by centrifugation at 8000 rpm for 10 min at 4°C. The artificial blood meal contained two parts freshly-harvested, clarified CxFV Izabal in cell culture supernatant, two parts defibrinated chicken blood (washed 3× in PBS), and one part FBS+10% sucrose, warmed to 37°C. *Culex quinquefasciatus* Sebring mosquitoes were allowed to feed for 30 min from a Hemotek feeder (Discovery Workshops, Accrington, Lancashire, UK). All unfed and partially fed mosquitoes were removed and discarded. An aliquot of the infected blood meal was reserved and held at 37°C during the length of the feed, then stored at −80°C for titration. Three to five mosquitoes were harvested at 0, 2, 4, 8, 12, and 14 DPI and processed as described above.

The effect of CxFV Izabal infection on WNV growth in cell culture and in mosquitoes was also determined. In cell culture, C6/36 cells were inoculated as above with CxFV Izabal at an MOI of 0.1. At 2 DPI, the supernatant was removed, cells were washed 3× with PBS and infected with WNV at an MOI of 0.1. WNV was adsorbed for 1 hour at 28°C. Cells were washed with PBS and 5 mL DMEM maintenance medium replaced. Concurrently, a control flask uninfected with CxFV Izabal was inoculated with WNV in the same manner. An aliquot of supernatant was harvested from each flask on Days 0, 2, 4, 6, 8, 10, and 14 following WNV infection, and WNV titer determined by Vero cell plaque assay. For WNV growth in mosquitoes, *Cx. quinquefasciatus* Sebring mosquitoes were divided into three groups. The first group was inoculated intrathoracically with 3.3 log_10_ pfu CxFV Izabal, the second group was mock-inoculated with an empty glass capillary needle, and the third group was not inoculated. Seven days post inoculation, each group was administered a WNV-infectious blood meal of 6.3 log_10_ pfu/mL. Three to five mosquitoes were harvested on Days 0, 2, 4, 8, and 10 days post infection with WNV and processed as described above. For each time point, the average WNV titers in CxFV Izabal (+), CxFV Izabal (−) and mock-inoculated groups were compared by 2-tailed pairwise Student's t-tests at the 5% significance level, assuming unequal variances.

For each growth curve, mosquitoes were homogenized individually in 2 mL conical microcentrifuge tubes containing a single copper bb and 1 mL DMEM with 10% FBS. Mosquitoes were ground for 4 min at 20 cy/s on a mixer mill MM300 (Retsch, Haan, Germany). Homogenates were clarified by centrifugation at 8,000 rpm for 10 minutes at 4°C. Supernatants were stored at −80°C until virus quantification.

### Vector competence for WNV

The ability of WNV to infect, disseminate, and be transmitted by *Cx. quinquefasciatus* infected with CxFV Izabal was evaluated across multiple WNV blood meal titers, routes of exposure to WNV, strains of *Cx. quinquefasciatus* mosquito, and prior infection with viable or inactivated CxFV Izabal. Artificial, infectious WNV blood meals were prepared as described above. In each experiment, one week-old *Cx. quinquefasciatus* were exposed to CxFV Izabal by intrathoracic inoculation with 2.8–3.3 log_10_ pfu seven days prior to receiving an artificial, WNV-infectious blood meal. Each CxFV Izabal (−) group was held, uninoculated, for one week and given the same WNV-infectious blood meal as the CxFV (+) group. In the first experiment, groups of Sebring strain *Cx. quinquefasciatus*, infected and uninfected with CxFV, received a WNV-infectious blood meal of 7.8 log_10_ pfu WNV per mL. In the second experiment, CxFV (+) and CxFV (−) Sebring and Honduras strain *Cx. quinquefasciatus* received WNV infectious blood meals of 8.9 log pfu per mL. In the third experiment, CxFV-positive and –negative Sebring and Honduras strain *Cx. quinquefasciatus* received a high titer (7.4–7.5 log pfu/mL) or low titer (5.4–5.6 log pfu/mL) WNV-infectious blood meal. Additional groups of mosquitoes were also inoculated with heat-inactivated (56°C for 45 min) CxFV Izabal to determine whether or not actively-replicating virus was necessary for any observed interference with WNV transmission.

For each WNV-infectious blood meal, an aliquot was reserved for plaque titration on Vero cells. Fully engorged mosquitoes were double-caged and held at 28°C at 95% relative humidity, and provided either 5% sucrose solution or raisins. At 14 days following the WNV-infectious blood meal, bodies, legs, and saliva were harvested from each live remaining specimen in each group and assayed for WNV by Vero cell plaque assay. Bodies and legs were each homogenized separately as described above. For saliva collections, specimens were knocked down by freezing at −20°C for 1 min, then, inside a glove box, wings were clipped off and the proboscis of each specimen was inserted into a capillary tube containing 5 µl Spectrosol immersion oil. After 20 min of salivation, specimens were removed from the capillary tube, and legs and bodies were separated into individual tubes. The tip of each capillary tube containing salivary expectorate was clipped off into a 1.7 mL microcentrifuge tube containing 450 µl DMEM with 10% FBS. Salivas were centrifuged for 5 minutes at 5000 rpm at 4°C to draw the oil out of the capillary tube, and stored at −80°C. The percentage of CxFV (+) and CxFV (−) mosquitoes that became infected, disseminated, and transmitted WNV were compared by Fisher exact test. The mean WNV titers in mosquito bodies and saliva 14 DPI between CxFV (+) and CxFV (−) experimental groups were compared by 2-tailed Student's t-tests assuming unequal variances.

### WNV transmission in co-inoculated specimens

To further evaluate WNV transmission in *Cx. quinquefasciatus* with a known WNV-disseminated infection, groups of *Cx. quinquefasciatus* Sebring and Honduras strain were inoculated with either WNV only or inoculated simultaneously with CxFV Izabal and WNV. Sebring specimens were inoculated with either 4.0 log_10_ pfu WNV (n = 66) or with 4.0 log_10_ pfu WNV+3.6 log_10_ pfu CxFV (n =  27) per mosquito. Honduras specimens were inoculated with either 3.9 log_10_ pfu WNV (n = 36) or 3.9 log_10_ pfu WNV+3.3 log_10_ pfu CxFV (n = 53) per mosquito. Nine days post inoculation, saliva was collected from each specimen as described above, and bodies were stored whole at −80°C. Salivary expectorates were analyzed as above.

## Results

### Virus detection and quantification

**Figure 1:**
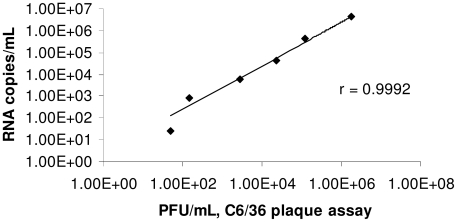
Correlation of CxFV Izabal qRT-PCR with C6/36 cell plaque titration. Samples from each dilution were split such that each dilution was quantified by both qRT-PCR and plaque assay. In this figure, the CxFV Izabal qRT-PCR assay used primers E92F and E151R and probe E112P. Results for other CxFV Izabal primer/probe sets are similar (data not shown).

Four novel quantitative RT-PCR (qRT-PCR) primer and probe sets were designed to amplify CxFV Izabal (Table 1). No amplification was obtained from WNV or KRV with any of the primer/probe sets. Primer and probe sequences were aligned to available genome sequences for CxFV [10] and CFAV to further examine their specificity for CxFV Izabal (Table 1). Correlation between CxFV Izabal qRT-PCR and C6/36 plaque assays was >99% (r = .9992). The equation for the trendline fit to the data was y = 2.47x, with the y-intercept fixed at zero (Fig. 1).

### Growth curves

**Figure 2:**
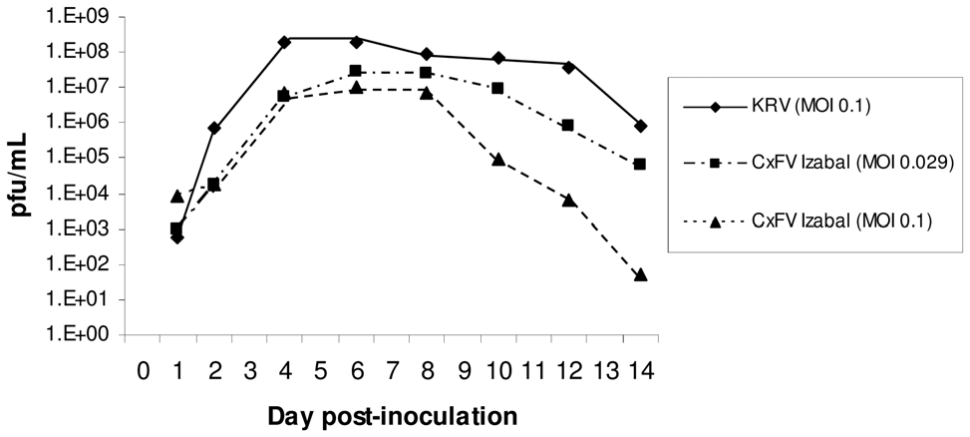
Replication of CxFV Izabal and KRV in C6/36 cells.

**Figure 3:**
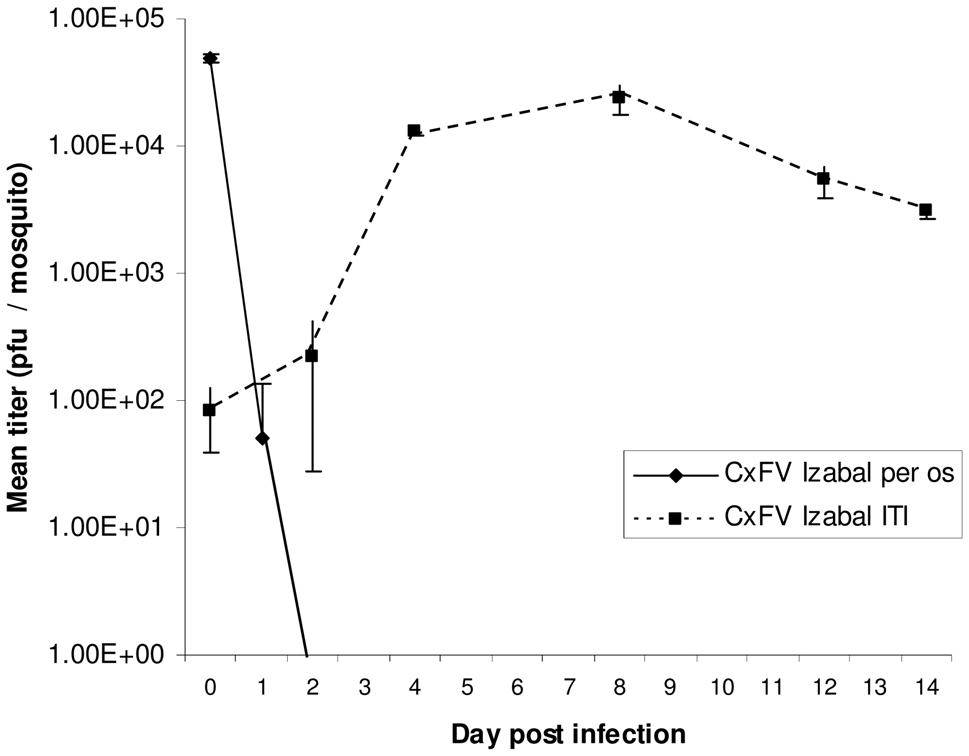
Replication of CxFV Izabal in *Cx. quinquefasciatus* Sebring mosquitoes. Values at each time point represent the mean ± standard error of three to five mosquitoes.

**Figure 4:**
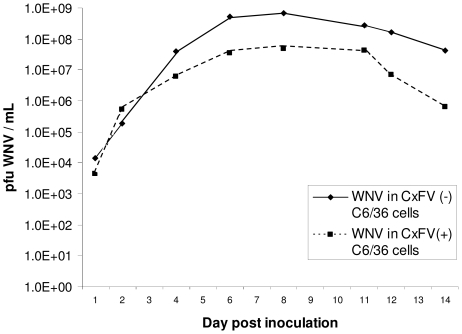
Replication of WNV in CxFV Izabal (+) and CxFV Izabal (−) C6/36 cells. CxFV (+) cells were infected two days prior to re-infection with WNV.

**Figure 5:**
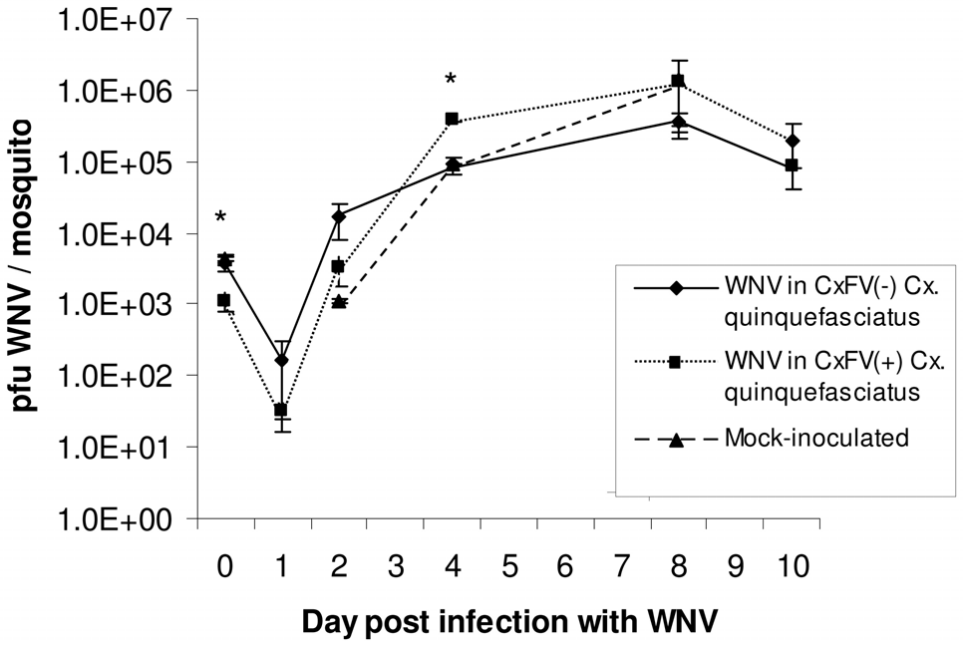
Replication of WNV in CxFV Izabal (+) and CxFV Izabal (-) *Cx. quinquefasciatus* Sebring mosquitoes. CxFV Izabal (+) mosquitoes were inoculated intrathoracically with 3.3 log_10_ pfu CxFV Izabal. Mock-inoculated mosquitoes were inoculated with an empty glass capillary needle, and CxFV (-) mosquitoes were not inoculated. Seven days post inoculations, all three groups were administered a WNV-infectious blood meal of 6.3 log_10_ pfu/mL. The average WNV titer in CxFV Izabal (+) mosquitoes was significantly less than in mock-inoculated specimens at 0 DPI (Student's t-test, p = 0.016), denoted by an asterisk. At 4 DPI the average WNV titer in CxFV Izabal (+) mosquitoes was significantly higher than in CxFV (-) specimens (Student's t-test, p = 0.0048), denoted by an asterisk. Values at each time point represent the mean ± standard error of two to five mosquitoes.

Replication kinetics of CxFV Izabal were determined in C6/36 cells and in *Cx. quinquefasciatus* Sebring strain mosquitoes (Figs. 2,3). Replication of WNV was also monitored in CxFV Izabal (+) and (−) C6/36 cells, and in CxFV Izabal (+) and (−) *Cx. quinquefasciatus* Sebring (Figs. 4,5). In C6/36 cells, CxFV Izabal (passage 1) reached a maximum titer of approximately 7.0 log_10_ plaque forming units (pfu)/mL six days following infection at either multiplicity of infection (MOI) of 0.03 or MOI = 0.1, approximately one log less than that observed for KRV (Fig. 2). CxFV Izabal caused evident cytopathic effects (CPE) in C6/36 cells, completely destroying the cell monolayer by 8 days post infection (DPI) following inoculation at an MOI of 0.1. In *Cx. quinquefasciatus* Sebring mosquitoes exposed to CxFV Izabal by intrathoracic inoculation, CxFV Izabal reached a peak titer of 4.3 log_10_ pfu/mosquito approximately 8 DPI. Mosquitoes were not susceptible to CxFV Izabal infection following oral exposure (Fig. 3).

Growth of WNV was not inhibited by CxFV Izabal in either C6/36 cells or *Cx. quinquefasciatus* Sebring mosquitoes (Figs 4,5). WNV titers were not significantly different between CxFV Izabal (+), CxFV Izabal (−) or mock-inoculated *Cx. quinquefasciatus* at 1, 2, 8, or 10 days following *per os* infection with WNV (p>0.05) (Fig. 5). At 0 DPI the average WNV titer in CxFV Izabal (+) mosquitoes was significantly less than in the mock-infected group (p = 0.016). It is unclear why this might be since mosquitoes were harvested immediately post-feeding and all three treatment groups imbibed the same WNV-infectious blood meal. At 4 DPI the average WNV titer in the CxFV Izabal (+) group was significantly higher than in the CxFV (−) mosquitoes (p = 0.0048) (Fig. 5). The biological significance of this observation is unclear, as this difference disappeared at 8 and 10 DPI (Fig 5). It is probable that this difference between groups is an artifact of small sample sizes (3–5 mosquitoes per time point).

### Vector competence for WNV: Sequential infections

The percentage of CxFV (+) and CxFV (−) *Cx. quinquefasciatus* that became infected, developed a disseminated infection, and transmitted WNV were not significantly different for either mosquito strain or any WNV blood meal titer examined (Fisher Exact test, p>0.05, Table 2). Furthermore, WNV infection, dissemination, and transmission rates in mosquitoes inoculated with heat-inactivated CxFV Izabal did not differ significantly from those inoculated with live CxFV Izabal or uninfected with CxFV Izabal (Table 2). There was extensive variation in WNV body and saliva titers in each of these groups (Table 3). West Nile virus titers in mosquito bodies and salivary expectorates were not significantly different between CxFV Izabal (+) and CxFV Izabal (−) *Cx. quinquefasciatus* when mosquitoes were exposed orally to WNV (Table 3, Student's t-test, p>0.05). One group of Sebring *Cx. quinquefasciatus* and one group of Honduras F12 *Cx. quinquefasciatus* failed to become infected (Table 2). We speculate that these results were not due to experimental treatment, but rather to small sample sizes and the relatively low WNV titer in those particular blood meals, potentially approaching a threshold of infection of approximately 5 log_10_ pfu WNV/mL [30], [31].

**Table 2:** WNV Infection, dissemination, and transmission rates in *Culex quinquefasciatus* either infected sequentially with CxFV Izabal and WNV, or uninfected with CxFV Izabal.

*Culex quinquefasciatus* strain	N	CxFV infection status	WNV blood meal titer (log_10_ pfu/mL)	Infection rate^1^	Dissemination rate^2^	Transmission rate^3^	Disseminated transmission rate^4^
Sebring	53	−	8.9	100	98	69	71
Sebring	28	+	8.9	100	100	57	57
Sebring	22	−	7.8	91	86	41	47
Sebring	14	+	7.8	79	64	36	56
Sebring	18	−	7.4	100	100	83	83
Sebring	7	+	7.4	86	86	71	83
Sebring	23	inactivated CxFV	7.4	96	96	83	86
Sebring	13	−	5.6	69	62	46	75
Sebring	10	+	5.6	60	50	20	40
Sebring	10	inactivated CxFV	5.6	0	0	0	0
Honduras F5/6	7	−	8.9	100	86	71	71
Honduras F5/6	8	+	8.9	100	100	75	75
Honduras F12	6	−	7.5	100	100	67	67
Honduras F12	2	+	7.5	100	100	50	100
Honduras F12	9	−	5.4	33	22	11	50
Honduras F12	6	+	5.4	0	0	0	0
Honduras F12	4	inactivated CxFV	5.4	75	50	0	0

Mosquitoes were exposed orally to WNV seven days post inoculation with CxFV Izabal. All comparisons between CxFV (+) and CxFV (−) groups were not significant (Fisher's Exact Test, p>0.05).

1 percentage of mosquitoes with WNV in their body 14 DPI.

2 percentage of mosquitoes with WNV in their legs 14 DPI.

3 percentage of mosquitoes with WNV in their saliva 14 DPI.

4 percentage of mosquitoes with a disseminated infection that contained WNV in their saliva 14 DPI.

**Table 3:** Mean WNV titer in *Cx. quinquefasciatus* bodies and salivary expectorates of specimens either infected or uninfected intrathoracically with CxFV Izabal.

*Culex quinquefasciatus* strain	N	CxFV infection status	WNV blood meal titer (log_10_ pfu/mL)	Mean WNV body titer (log_10_ pfu/mosquito ±SE)	Mean WNV saliva titer (log_10_ pfu/expectorate±SE)
Sebring	53	−	8.9	7.1±6.3	4.1±3.8
Sebring	28	+	8.9	7.1±6.3	4.6±4.4
Sebring	22	−	7.8	7.1±6.6	4.6±4.2
Sebring	14	+	7.8	6.5±6.3	4.3±3.9
Sebring	18	−	7.4	7.0±6.3	4.4±4.0
Sebring	7	+	7.4	6.8±6.5	4.7±4.5
Sebring	23	inactivated CxFV	7.4	7.0±6.5	3.9±3.4
Sebring	13	−	5.6	6.8±6.3	4.4±4.4
Sebring	10	+	5.6	6.4±6.3	3.8±3.8
Sebring	10	inactivated CxFV	5.6	-	-
Honduras F5/6	7	−	8.9	7.1±6.6	3.6±3.6
Honduras F5/6	8	+	8.9	6.9±6.2	2.8±2.5
Honduras F12	6	−	7.5	6.7±6.1	3.7±3.5
Honduras F12	2	+	7.5	6.7±0.0*	2.7±0.0^1^
Honduras F12	9	−	5.4	6.4±6.4	2.9±0.0^1^
Honduras F12	6	+	5.4	-	-
Honduras F12	4	inactivated CxFV	5.4	6.2±6.2	-

Bodies and salivas were harvested 14 days following an infectious WNV blood meal. Means were calculated from those bodies or salivas that tested WNV (+). Neither body titers or saliva titers were significantly different between CxFV (+) and CxFV (−) groups (Student's t-test, p>0.05).

1 only one sample so SE could not be calculated.

### Simultaneous infections

**Figure 6:**
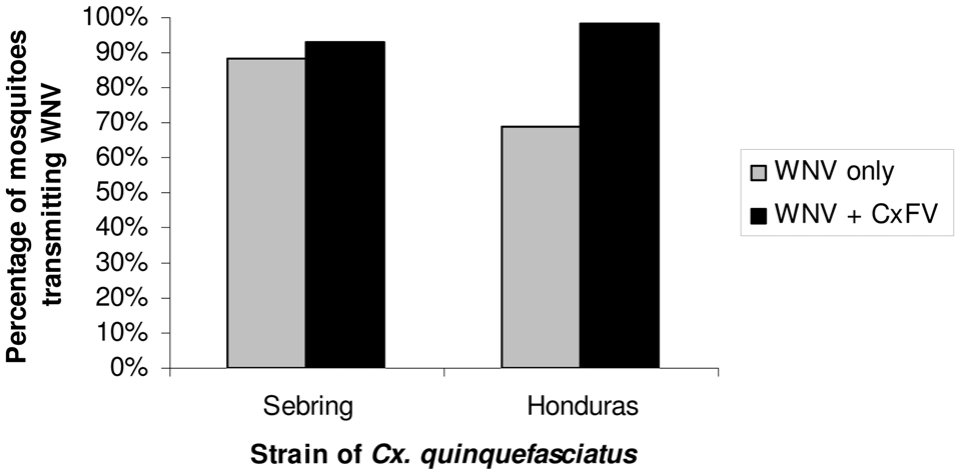
WNV transmission by CxFV Izabal (+) and CxFV Izabal (-) mosquito colonies. Percentage of *Cx. quinquefasciatus* transmitting WNV nine days following intrathoracic inoculation with either WNV alone or WNV + CxFV Izabal. For the Honduras colony, a significantly higher percentage of mosquitoes inoculated simultaneously with both viruses transmitted WNV than mosquitoes that were inoculated with WNV alone (Fisher's exact test, p = 0.0014).

**Figure 7:**
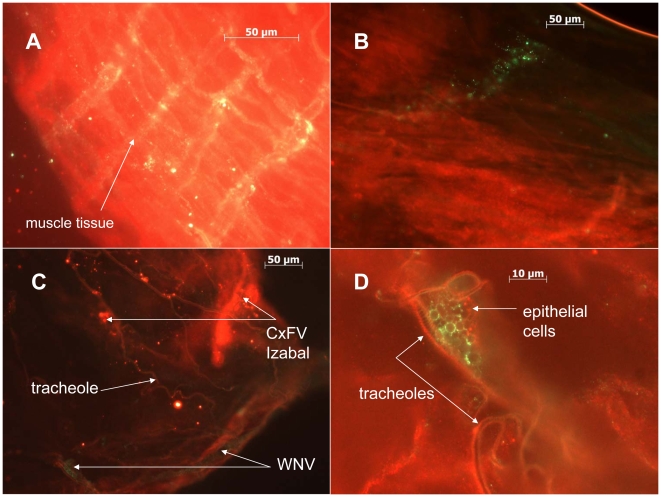
Localization of CxFV Izabal and WNV to the midgut in co-infected mosquitoes. A) WNV-infected muscle tissue of *Cx. quinquefasciatus* Sebring, harvested 9 DPI. WNV stained with AlexaFluor 488 (green). B) Focus of CxFV Izabal infection on the midgut of *Cx. quinquefasciatus*. CxFV Izabal stained with AlexaFluor 488. C) Midgut of *Cx. quinquefasciatus* infected simultaneously with both WNV and CxFV Izabal by intrathoracic inoculation. CxFV Izabal stained with AlexaFluor 594 (red) and WNV with AlexaFluor 488 (green). D) Close-up view of co-infected midgut from panel C showing midgut epithelial cells infected with WNV and CxFV Izabal. CxFV Izabal stained with AlexaFluor 594 (red) and WNV with Alexa 488 (green).

**Figure 8:**
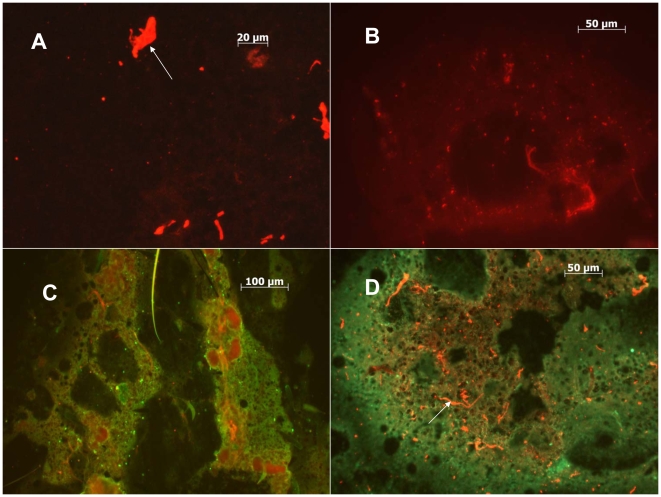
Localization of CxFV Izabal and WNV to head tissues in co-infected mosquitoes. A) Uninfected head tissues of *Cx. quinquefasciatus* stained with AlexaFluor 594 (red). White arrow depicts non-specific staining of debris. B) Head tissues of CxFV Izabal-infected *Cx. quinquefasciatus*, harvested 7 DPI. CxFV Izabal stained with AlexaFluor 594. C) Head tissues of WNV-infected *Cx. quinquefasciatus*, harvested 9 DPI. WNV stained with AlexaFluor 488 (green). D) Co-infected head tissues of *Cx. quinquefasciatus*. Mosquito inoculated simultaneously with CxFV Izabal and WNV, harvested 9 DPI. CxFV Izabal stained with AlexaFluor 594 (red) and WNV with Alexa 488 (green). White arrow denotes non-specific staining of debris.

A significantly higher percentage of Honduras *Cx. quinquefasciatus* transmitted WNV when co-inoculated simultaneously with CxFV Izabal (98%, n = 53) than when inoculated with WNV alone (69%, n = 36) (p = 0.0014, Fisher Exact test) (Fig. 6). The percentage of Sebring *Cx. quinquefasciatus* that transmitted WNV when co-inoculated simultaneously with CxFV Izabal (93%, n = 27) was not significantly different from those inoculated with WNV alone (88%, n = 66) (p>0.05, Fisher exact test) (Fig. 6). The percentage of intrathoracically-inoculated specimens that transmitted WNV alone was also significantly less in the Honduras colony as compared with the Sebring colony, suggesting a more effective salivary gland barrier to WNV in the Honduras colony (p = 0.033, Fisher exact test); 87% of Sebring specimens (n = 66) transmitted WNV compared with only 69% (n = 36) of the Honduras specimens. For the Sebring colony, the average WNV titer in salivary expectorates for specimens inoculated with WNV only was 4.4 log_10_ pfu (n = 58), and not significantly different from an average titer of 4.7 log_10_ pfu in the expectorates of WNV+CxFV Izabal group (n = 25) (Student's two-tailed t-test, p = 0.11). For the Honduras colony, the average WNV titer in salivary expectorates for specimens inoculated with WNV only was 4.6 log_10_ pfu (n = 25) compared with 4.8 log_10_ pfu in the WNV+CxFV Izabal group (n = 52) (Student's two-tailed t-test, p = 0.38). For these groups, co-inoculated mosquitoes that transmitted WNV also contained CxFV Izabal in their saliva and mosquitoes that did not transmit WNV also did not transmit CxFV (n = 12). Mosquitoes infected with CxFV Izabal only (n = 5) did not have CxFV Izabal in their saliva. Midgut (Fig. 7) and head tissues (Fig. 8) of mosquitoes inoculated simultaneously with CxFV Izabal and WNV were observed to be infected with both viruses by IFA.

## Discussion

In this study we demonstrated that sequential infection of C6/36 cells or *Cx. quinquefasiatus* mosquitoes with CxFV Izabal and WNV did not interfere with either growth or transmission of WNV. This finding is not surprising given that Culex flaviviruses are being discovered in mosquito populations around the world in locations where WNV and other flaviviruses circulate sympatrically. Therefore, the prevalence of CxFV Izabal in *Cx. quinquefasciatus* in Guatemala does not explain the lack of human disease attributable to WNV in this region.

Growth of WNV in C6/36 cells was not inhibited by prior infection of CxFV Izabal. The WNV titer in CxFV Izabal (+) C6/36 cells did not reach the maximum titer observed in CxFV Izabal (−) cells due to death of cells caused by CxFV Izabal (Fig. 4). As suggested by Hoshino et al. [10], CPE observed in C6/36 cells may be the result of an unnatural association between this *Culex*-derived virus and *Aedes*-derived cell line since CxFV apparently replicates avirulently in its mosquito host. Therefore, future studies should include utilization of *Culex*-derived cell lines. The natural host range of CxFV Izabal across mosquito species and genera is not known.

Data regarding the establishment of superinfection by homologous viruses in cell culture have been variable. C6/36 cells persistently infected with *Aedes aegypti* densonucleosis virus remained permissive to infection with *Haemagogus equinus* densovirus (HeDNV), arguing against the induction of an anti-viral or immune state in the cells that would otherwise inhibit superinfection by this a similar virus [32]. However, interference between superinfecting alphaviruses in mosquito cell culture has been documented multiple times [22]–[24]. The cellular and molecular mechanisms that support replication of WNV in CxFV (+) cells are not known and require further study.

Overall, neither growth nor transmission of WNV in *Cx. quinquefasciatus* was significantly affected by CxFV Izabal when viruses were administed sequentially. These findings are in contrast to what has been found previously for flavivirus - flavivirus superinfections involving WNV in *Culex* mosquitoes, however insect-only flaviviruses are fairly divergent from other vector-borne flavivirues such as WNV [5], [10], [33]. Previous studies have demonstrated that transmission of a superinfecting flavivirus was blocked if the secondary flavivirus was antigenically-similar to the primary infecting flavivirus [17], [18]. Interference to arbovirus superinfection in mosquitoes or mosquito cells by homologous viruses could be the result of RNA interference (RNAi). RNAi is a mechanism by which invertebrates respond to viral infection through the specific recognition and degradation of viral mRNA sequences by virus-derived small interfering RNAs (viRNA) [34]–[38]. However, our data demonstrate that prior infection with CxFV Izabal does not interfere with WNV replication when the viruses are inoculated simultaneously, or when mosquitoes are exposed to WNV one week following inoculation with CxFV Izabal. If an RNAi pathway was induced in *Cx. quinquefasciatus* by CxFV Izabal it would most likely still be effective seven days post-inoculation when mosquitoes were exposed to WNV as it has been previously reported that viRNAs targeting Sindbis virus in C6/36 cells were first detected 48h following infection and were still abundant 7 DPI [38], and viRNAs targeting WNV in *Cx. quinquefasciatus* midguts were detected 7 and 14 days post exposure to WNV [37].

There are numerous potential reasons why mosquitoes would be permissive to co-infecting flaviviruses. First, if CxFV Izabal induced an RNAi response in *Cx. quinquefasciatus*, the viRNAs generated might not be sufficiently homologous to WNV to interfere with the establishment of WNV infection. RNAi is a highly sequence-specific mechanism with little tolerance to mismatches between the viRNA trigger and mRNA target sequences [39], and the nucleotide sequence identity between CxFV and other vertebrate-infecting flaviviruses is relatively low. Kim et al. [13] reported between 25 and 52% nucleotide sequence homology between CxFV (TX24518) and WNV among structural and non-structural genes. Similarly, Hoshino et al. [10] reported 17–25% sequence identity for structural proteins and 17–40% identity among non-structural proteins between their Japanese isolate of CxFV and other flaviviruses. Therefore, any interference of CxFV Izabal viRNAs with WNV was probably minimal. Secondly, it is possible that over a history of co-evolution between insect-only flaviviruses and their mosquito hosts, these viruses have evolved a way to either evade or suppress an immune mechanism that would otherwise interfere with their own replication, or replication of a subsequently-infecting virus. Flock house virus encodes an RNAi suppressor protein, B2, that is necessary for establishment of viral infection in Drosophila S2 cells [40]. Virus-encoded suppressors of RNAi have also been found in plant viruses such as tobacco etch potyvirus [41]. The molecular mechanisms that permit co-existence of both WNV and CxFV Izabal, potentially even within the same tissues and cells, requires further study. Thirdly, CxFV replication in mosquito cells is presumably similar to that of other flaviviruses due to similar genome organization [10], [13]. Zebovitz and Brown [22] determined that interference of superinfecting alphaviruses in cell culture was due to competition for replication sites or metabolites, and that viral RNA synthesis was necessary for inhibition of alphavirus superinfection. The non-structural proteins encoded by flaviviruses play important roles in virus replication and maturation [42], and the 5′ and 3′ untranslated regions contain conserved nucleotide sequences and RNA secondary structures involved in virus replication and translation [43]. Camissa-Parks et al. [44] discovered that the 3′ stem loop structure of CFAV differed from that of other vertebrate-infecting flavivirus RNAs, and the 3′ pentanucleotide sequence, which is completely conserved among mosquito- and tick-borne flaviviruses contained a point mutation in cell fusing agent virus. The function of this pentanucleotide sequence element is thought to be involved in the binding of cellular or viral proteins to the 3′ stem loop structure during RNA replication [43]. The 3′ UTR of CxFV also was found to contain four tandem repeats, hypothesized to be specially adapted for replication in the mosquito host [10] since deletion of conserved tandem repeat sequences alters virus growth properties [43]. Finally, CxFV may target and replicate in different mosquito tissues than WNV or other flaviviruses. In *Culex quinquefasciatus* inoculated simultaneously with CxFV Izabal and WNV, midgut and head tissues became infected with both viruses, demonstrating a potential for physical interaction between CxFV Izabal and WNV (Figs. 7,8). However it is unclear if these tissue tropisms would be the same for mosquitoes naturally-infected with CxFV or if infection of these tissues is an artifact of inoculation, or inoculation simultaneously with WNV. More work is needed on characterizing the tissue tropisms of CxFV in naturally-infected mosquitoes and the mechanism by which this virus propagates and is transmitted.

One limitation of this study is that the natural mechanism by which mosquitoes become infected with CxFV has not yet been elucidated, so mosquitoes in this study were infected with CxFV Izabal by intrathoracic inoculation. It is unclear how or if the results of this study would be different using naturally-infected mosquitoes. Route of infection has been shown to affect the outcome of arbovirus superinfection studies. Most notably, *Aedes triseriatus* mosquitoes that were infected transovarially with LaCrosse virus remained permissive to secondary infection with a homologous or heterologous bunyavirus [45], whereas mosquitoes exposed to the primary infection by intrathoracic inoculation became refractory to superinfection after seven days [20]. Ideally, these studies should be repeated using mosquitoes naturally-infected with CxFV to fully understand the dynamics of interaction, or lack thereof, between these two flaviviruses within the mosquito vector. However the advantage of inoculations is that experiments can be standardized by infecting mosquitoes of the same age with approximately the same amount of virus, and 100% infection rates are assured.

Interestingly, both CxFV Izabal and WNV were found in saliva of co-infected specimens when mosquitoes were exposed to both viruses simultaneously by intrathoracic inoculation, but no CxFV Izabal was found in the saliva of singly-infected specimens. This observation suggests that CxFV Izabal may be infecting the salivary glands by “piggybacking”on WNV. This phenomenon has been suggested for expansion of cellular tropism by human immunodeficiency virus (HIV), whereby Epstein-Barr virus, cytomegalovirus, human t-lymphotrophic virus, and sperm proteins share large regions of similarity with the CD4 protein of T-helper lymphocytes, a cellular receptor used by HIV [46]. Because HIV binds to CD4, binding of HIV to CD4 homologues on other co-infecting viruses or sperm may allow HIV to “piggyback” into additional cell types which it normally would not infect [46]. The molecular basis for this interaction between CxFV Izabal and WNV and how these results compare to natural infection is unknown. Our intrathoracic inoculation data suggest that CxFV Izabal may have the potential to enhance WNV transmission in some mosquito populations; however WNV transmission was not enhanced in Honduras colony when mosquitoes were exposed *per os* (Table 2).

In summary, this is the first study to address the potential effect of an insect-specific flavivirus on transmission of WNV. We have demonstrated that CxFV Izabal does not interfere with growth of WNV in C6/36 cells or in *Cx. quinquefasciatus*, nor does it inhibit infection, dissemination, or transmission of WNV. These findings are in contrast to what would be expected based on previous studies following flavivirus – flavivirus superinfections. We hypothesize that both CxFV Izabal and WNV have evolved mechanisms for persistence and transmission by a common mosquito vector, *Cx. quinquefasciatus*, despite the presence of mosquito immune defenses and the prevalence of co-circulating flaviviruses. Future studies should address the effect of CxFV and WNV co-infection in mosquitoes naturally infected with CxFV, as well as the tissue tropisms and molecular mechanisms of CxFV replication and transmission in mosquitoes.
